# A New Surface Plasmon Resonance Immunosensor for Triazine Pesticide Determination in Bovine Milk: A Comparison with Conventional Amperometric and Screen-Printed Immunodevices

**DOI:** 10.3390/s150510255

**Published:** 2015-04-30

**Authors:** Mauro Tomassetti, Elisabetta Martini, Luigi Campanella, Gabriele Favero, Gabriella Sanzó, Franco Mazzei

**Affiliations:** 1 Department of Chemistry, Sapienza University of Rome, P. Le Aldo Moro 5, 00185 Rome, Italy; E-Mail: luigi.campanella@uniroma1.it; 2 Department of Chemistry and Drug Technologies, Sapienza University of Rome, P. Le Aldo Moro 5, 00185 Rome, Italy; E-Mails: gabriele.favero@uniroma1.it (G.F.); gabriella.sanzo@uniroma1.it (G.S.); franco.mazzei@uniroma1.it (F.M.)

**Keywords:** SPR, immunoassay, triazine pesticide analysis, bovine milk

## Abstract

A detailed comparison was made of the analytical features of a new Surface Plasmon Resonance (SPR) immunodevice for triazine pesticide determination with those of two other amperometric (conventional and screen-printed) immunosensors and the advantages and disadvantages of the SPR method were thoroughly investigated. For conventional amperometric and screen-printed devices, “competitive” assays were used; conversely, the SPR transduction technique allowed a “direct” measurement format to be used. As far as the main analytical data are concerned, the SPR method does not seem to offer substantial advantages. Nevertheless the measurement time is much shorter and the measurement itself much easier to perform. Lastly several applications and recovery tests were carried out on bovine milk samples, before and after spiking, to check for triazine pesticides in the samples, obtaining satisfactory results.

## 1. Introduction

The extensive use of pesticides for agricultural and non-agricultural purposes means that it is almost impossible to avoid daily exposure to a number of pesticides contained in both food and water [1,2,3,4]. The triazine herbicides are among the most widely used weedkillers, as triazine herbicides such as atrazine, simazine and other triazine compounds are currently applied to corn, wheat, barley and legumes and several fruit crops for the purpose of broadleaf weed control. The European Community Directive sets a maximum admissible concentration of 0.1 µg·L^−1^ for any single herbicide in water intended for human consumption. In USA, the Environmental Protection Agency (EPA) allows an upper limit of 3.0 µg·L^−1^ for atrazine in drinking water. Most often, triazine residues are analyzed by chromatographic techniques, such as thin layer chromatography [5,6], gas-chromatography (GC) [7,8], liquid-chromatography (LC), or mass-spectrometry (MS) [9,10].

These methods are undoubted highly sensitive and routinely used in the chemical laboratory, but nevertheless these important techniques are not cheap, and they are time-consuming and not suitable for on-site analysis, as they generally require sample pre-treatment and involve complex and expensive equipment [5,6,7,8,9,10,11]. On the contrary, biosensor methods (and thus also immunosensors) are usually rapid, inexpensive and suitable for *in situ* analysis [12,13,14,15,16,17]. Therefore several enzymatic electrode-based methods for pesticide detection have been developed which utilize the inhibition of an enzyme, such as tyrosinase [18] or butyrylcholinesterase [19]. Our research group has worked on inhibition biosensors, in particular tyrosinase biosensors, designed to analyse triazine and benzotriazine compounds [19,20]. In recent years, our team has investigated the development of conventional immunosensors [21,22,23,24], or screen-printed methods [25,26] above all because they were found to be more selective for the different classes of pesticide. In this framework the aim of the present research was to compare the analytical features of a new SPR device with two conventional or screen-printed amperometric immunosensor devices. The k_aff_ values were also evaluated and compared. Lastly, these immunosensors were used to test triazine pesticides and to apply recovery tests from common real matrices such as several bovine milk samples.

## 2. Results

### 2.1. SPR Immunodevice Assembly and Direct Flow Measurements 

**Figure 1:**
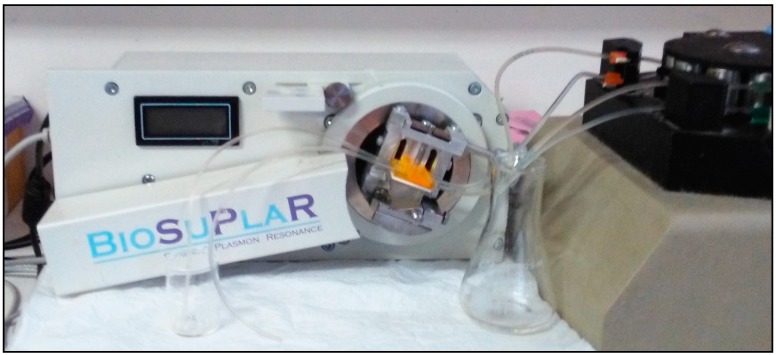
Measurement instrument used for Surface Plasmon Resonance operating in flow mode.

This flow SPR device (see Figure 1) was assembled using Kretschmann geometry for excitation of plasmons on the gold surface. The main element of the device is the retroreflecting measurement prism, which is installed on a rotating table that is controlled automatically by a computer. The maximum operational angle possible is 17°. The different samples are pumped through a flow cell realized on the gold sensor placed over the prism surface and the flow was controlled by a peristaltic pump. A polarized light was focused at the surface of the sensor and the angular dependence of the reflected light was recorded. Modification of the Au sensing surface was carried out as follows: a self-assembled monolayer was obtained by immersing the Au disk in an ethanol solution containing 2 mM of 1,1-mercaptoundecanoic acid (MUA) in order to obtain a self-assembled monolayer (SAM). After 12 h, the disk was rinsed with ethanol, dried by a nitrogen stream and placed on the SPR prism using a standard refractive index oil. Then, the instrument assembly was completed with a cell intended for flow analysis which was pressed on the Au layer. To observe the SPR phenomenon, the intensity of the polarized light reflected by the Au layer was investigated as a function of the angle of incidence, upon moving the table with the prism in order to change the angle of incidence. In order to re-hydrate the sensor, a 10 mM phosphate buffer solution pH 8.0 was allowed to flow for 1 h. After the sensor became stable, a solution containing 0.5 mM of ethyl(dimethylaminopropyl) carbodiimide (EDC) and 0.1 mM N-hydroxysuccinimide (NHS) was allowed to flow for 15 min in order to activate the SAM carboxylic groups. The disk was rinsed with phosphate buffer and a 10^−6^ M solution of anti-atrazine was allowed to flow for 40 min to achieve a covalent bond between the carboxylic group and the antibodies. Then a solution of 10^−3^ M ethanolamine was utilized for 15 min to deactivate carboxylic groups thus reducing non-specific adsorptions. The analysis of atrazine was performed by measuring the SPR resonance angle upon injection of various standard solutions thereof. In particular, after a baseline was obtained by flowing 10 mM phosphate buffer pH 8.0, a solution of atrazine was allowed to flow into the cell and the relative signal increase was observed. Successively, when a plateau was reached, phosphate buffer was allowed to flow in order to eliminate the atrazine that was not bound to the anti-atrazine. The regeneration of the surface was realized by allowing a 0.5 M NaCl, pH 2.5 solution to flow in the cell that dissociate the complex between anti-atrazine and atrazine, then when the signal has reached a new baseline, a new measurement was performed.

### 2.2. Conventional and Screen-Printed Immunosensors Assembly 

**Figure 2:**
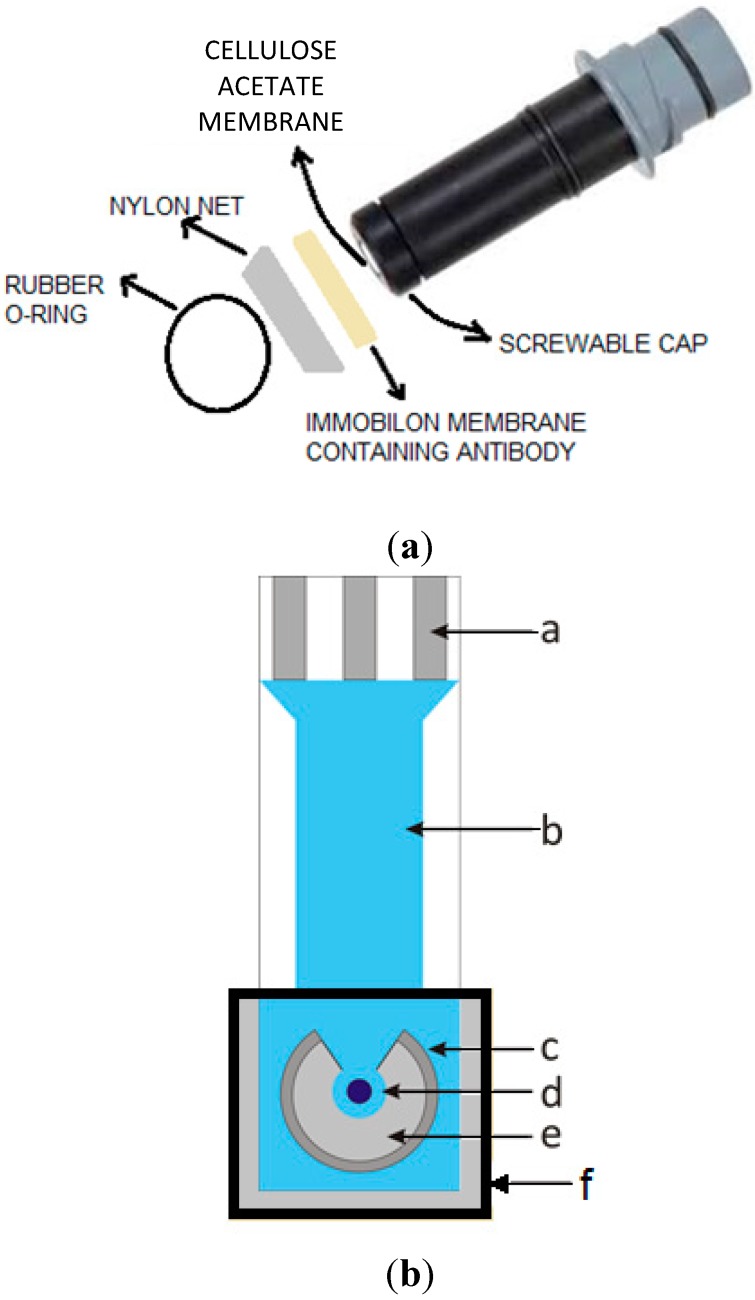
View of (**a**) conventional amperometric immunosensor and (**b**) the screen-printed electrode, both used for atrazine determination: **a**: electrical connections; **b**: insulating ink; **c**: Pt counter electrode; **d**: Prussian Blue electrodeposited onto Pt working electrode; **e**: Ag/AgCl/Cl^−^ reference electrode; **f**: Immobilon membrane with immobilized antibody.

The conventional immunosensor was assembled (Figure 2a) using an Immobilon membrane in which the antibody was immobilized, overlapping a cellulose acetate membrane (0.1 mm thick) placed on the lower end of the plastic cap of an amperometric electrode for H_2_O_2_. A nylon net and an O­ring were used to fix the Immobilon membrane to the head of the cap itself. The transducer used consisted of a conventional amperometric electrode for hydrogen peroxide, provided with a plastic cap filled with a 0.1 M KCl solution screwed onto the body of the electrode. A constant potential of +650 mV, with respect to an Ag/AgCl/Cl^−^ reference electrode, was applied to the Pt anode. Horseradish peroxidase enzyme (HRP) was used as a marker for immunocomplex detection. The assembly of the screen-printed electrode is illustrated in Figure 2b using a Pt working electrode modified by Prussian Blue, overlapping an Immobilon membrane containing the immobilized antibody.

### 2.3. The Albumin–Atrazine Conjugation

Atrazine carboxy derivative [27] (20 mg), *N*,*N*­dicyclohexylcarbodiimide (18.6 mg) and *N*­hydroxysuccinimide (NHS, 10.5 mg) were dissolved in dimethylformamide (3 mL) and incubated under stirring for 4 h at room temperature. The NHS­activated atrazine thus obtained was immediately used for conjugation with BSA. Two hundred microlitres of the mixture were added to phosphate buffer (2 mL, 0.05 M, pH 7.0) containing BSA (10 mg) and incubated for 4 h at room temperature [24,28].

### 2.4. Albumin–Atrazine Biotinylation

The avidin–biotin peroxidase technique is based on the use of a biotinylated antigen or antibody and avidin horseradish peroxidase conjugate as part of the labelling system [29,30,31]. The technique exploits the high affinity binding of biotin to avidin. The BiotioTag kit is specially designed for the small scale labelling of antibodies using biotinamido hexanoic acid 3­sulfo­N­hydroxysuccinimide ester (BAC­SulfoNHS) as the labelling reagent. After the labelling reaction, the biotinylated protein is separated from the unreacted or hydrolyzed reagent in a fast gel­filtration step using G­50 microspin columns. BAC­SulfoNHS reacts with free amino groups of proteins to form stable amide bonds. Extravidin binds to biotin with a high affinity and specificity. The high affinity for biotin alleviates non­specific binding interactions commonly associated with the strongly basic avidin protein [29,30,31]. The use of the extended spacer arm greatly improves the interaction between extravidin and the biotinylated macromolecule, thus overcoming the steric hindrance present at the biotin binding sites of extravidin [32]. Detailed descriptions of this technique were reported in previous papers [23,24,25,26]. 

### 2.5. Antibody Immobilization on the Immobilon Membrane

The positively charged nylon Immobilon Ny^+^ membranes were cut into approximately 1 cm^2^ disks; then anti­atrazine solution (50.0 μL, 10 M) was prepared in an Eppendorf test tube by dissolving anti­atrazine (about 5 mg) in phosphate buffer (350 mL, 0.1 M, pH 7.0) and coated directly on to the surface disk membrane. The Immobilon membrane thus obtained was then dried at room temperature for about 24 h and stored at 4 °C.

### 2.6. Atrazine Determination with Competitive Format Using Conventional or Screen-Printed Devices

**Figure 3:**
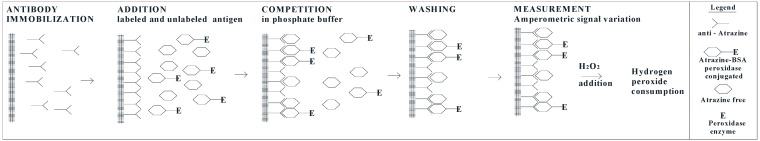
Measurement: competition between atrazine and a fixed concentration of peroxidase atrazine conjugated, both free in phosphate buffer solution, for the antibody immobilized on the membrane.

Test geometry: competition between atrazine and albumin–atrazine conjugated with biotin–avidin–peroxidase, both free in solution, for anti­atrazine immobilized in the membrane were the same as described in a previous papers [21,33]. Conventional and screen-printed immunosensors both used an amperometric electrode for hydrogen peroxide as transducer. Lastly different construction techniques were used, but in all cases horseradish peroxidase was used as enzymatic marker; furthermore the “competitive” immunological format was used in the two latter cases. Briefly, we developed both a screen-printed immunosensor and a conventional amperometric immunosensor; in both these cases the measurement method was based on the formation of labelled immunocomplex on a suitable (Immobilon) polymeric membrane of the immunosensor, after competitive assay between the free antigen and a fixed concentration of enzyme labeled antigen for the anti-atrazine immobilized on the membrane, horseradish peroxidase being the enzymatic marker. The competitive format used is illustrated in Figure 3.

The Immobilon membrane, on which the anti­atrazine was immobilized as described in the Section 2.5, was fixed to the head of the amperometric electrode for hydrogen peroxide. Before measurement, the immunosensor was dipped into a TRIS–HCl buffer solution, 0.1 M (pH 8.0), containing 0.05% Tween­20 by weight and 2.5% BSA by weight (bovine albumin was used to minimize non specific adsorption on the membrane).

To TRIS–HCl buffer solution (5 mL, 0.1 M, pH 8.0) renewed in the measurement cell, the atrazine sample to be determined was added together with a fixed concentration of peroxidase conjugated albumin–atrazine–biotin–avidin (50 mL, 10^−3^ M). For 1 h the enzyme­conjugated atrazine was allowed to compete with the non­conjugated atrazine free in solution in binding the anti­atrazine immobilized on the Immobilon membrane. After washing with the same buffer solution to remove all the unbound labelled atrazine, the specific substrate of the enzyme, *i.e.*, H_2_O_2_ solution (20 mL, 1% v/v), was added to the renewed buffer solution in which the immunosensor was dipped, under stirring. The enzymatic reaction was catalyzed by HRP enzyme bound to the enzymatic marker. The measured signal (nA) correlated directly with the atrazine concentration to be measured. In this case, hydrogen peroxide produced a signal that increased with increasing concentration of atrazine free in solution. A calibration curve was therefore constructed by plotting the amperometric signal (in nA) on a semilogarithmic scale as a function of increasing atrazine concentration in solution and used to determine the unknown concentration of atrazine contained in any sample whatever. The enzymatic reaction response took about 15–20 min. Individual measurements were performed, each time using a new membrane.

### 2.7. Selectivity and k_aff_ Measurements

Theoretically, the response of each immunosensor should be extremely selective owing to the specificity of the antigen-antibody reaction. However its selectivity must be verified experimentally because of the phenomenon of cross-reactivity, a situation in which adverse reactions occur when the immunosystem is deceived by a similar chemical compound. The antibody may react to atrazine antigens of the same family. Consequently, first of all, the selectivity was evaluated considering compounds structurally related to atrazine, that is other triazinic pesticides (simazine, atrazine-desethyl, azinphos-ethyl). To this end, a comparison was made between the slope values of calibration straight line, obtained by the immunosensor for each of these analytes in approximately the same concentration range. Then, by the same method, the selectivity toward non triazinic pesticides such as carbamate and the phenoxy family of herbicides, such as carbaryl and aldicarb, or 2,4-D was also checked. In addition, an estimation was made of the value of k_aff_ (affinity constant) found using the Langmuir curve, which is dependent on the concentration measurement at which half the maximum response (IC_50_) occurs [22,24,25].

### 2.8. Pesticide Measurement in Bovine Milk Samples and Recovery Tests

First of all immunosensors described in this paper were used to measure the triazine pesticides (atrazine, simazine, atrazine-desethyl, azinphos-ethyl) possibly contained in commercial bovine milk samples analyzed without any pretreatment using the respective calibration curves. Successive recovery tests were performed; to this end milk samples (0.5 mL) were added to phosphate buffer (4.5 mL) and the measurement was performed as described above in Section 2.1 and Section 2.6. Using the respective pesticide calibration curves to obtain the final concentration of pesticides. In addition, recovery tests were carried out on the milk samples spiked with known concentrations of each pesticide in order to obtain a final pesticide concentration of about 10^−7^ M. For this purpose a solution of pesticide (200 µL, 10^−4^ M) was dissolved in phosphate buffer (4.5 mL) to which commercial bovine milk samples (0.5 mL) were added. Also in this case the measurements were performed as described in Section 2.1 or Section 2.6, respectively, and using the respective calibration curves to obtain the concentrations of spiked solutions.

## 3. Discussion

**Figure 4:**
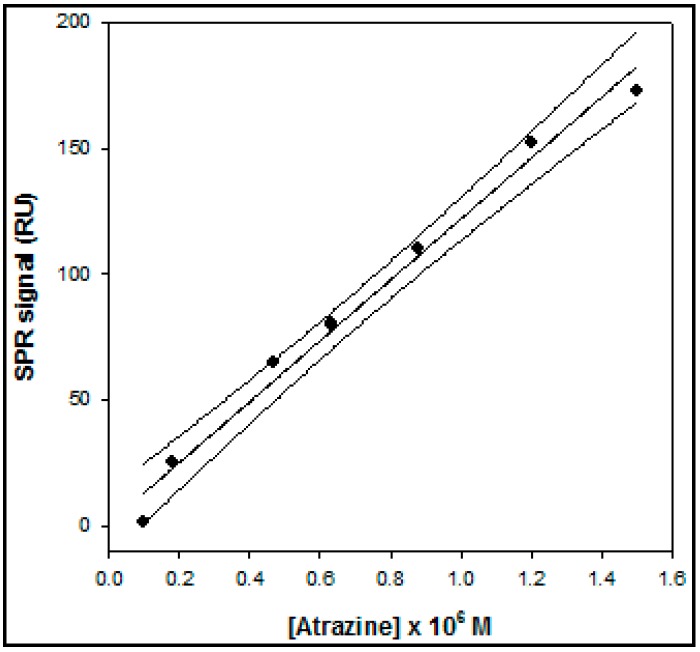
Calibration curve, obtained by the direct method, based on Surface Plasmon Resonance (SPR), operating in flow mode, for atrazine determination.

**Figure 5:**
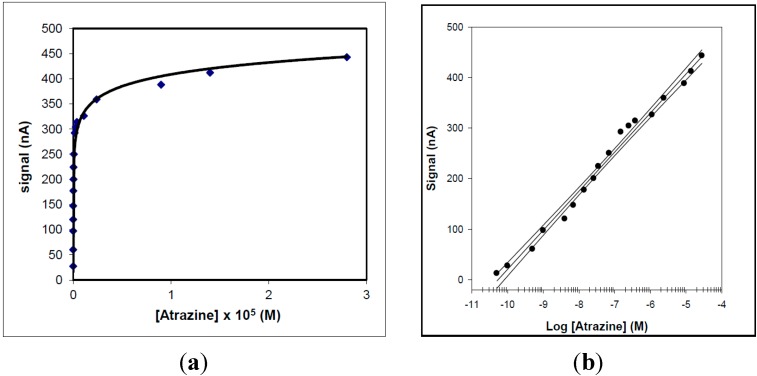
(**a**) Behaviour of the conventional amperometric immunosensor response as a function of increasing atrazine concentration, using an H_2_O_2_ electrode as transducer and peroxidase enzyme as marker; (**b**) corresponding calibration curve and confidence interval for atrazine determination, obtained using a semilogarithmic scale.

**Figure 6:**
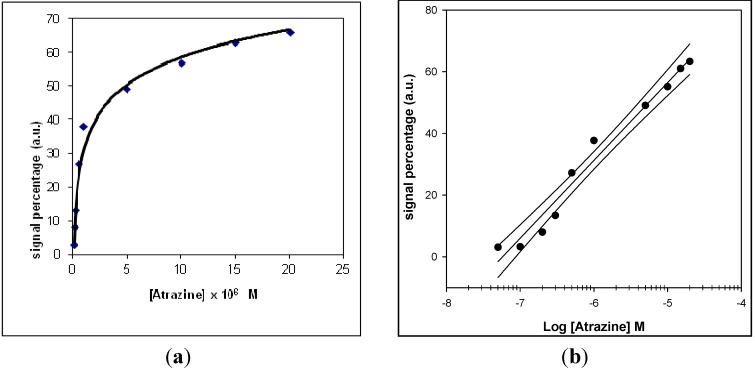
(**a**) Behaviour of the screen-printed immunosensor response as a function of increasing atrazine concentration, using peroxidase enzyme as marker and an amperometric electrode for H_2_O_2_ as transducer; (**b**) corresponding calibration curve and confidence interval for atrazine determination, obtained using a semilogarithmic scale.

In the case of the SPR device the calibration curve for atrazine (Figure 4) showed a linearity range of 1.0 × 10^−7^ to 1.5 × 10^−6^ M and with a LOD value of approximately 5 × 10^−8^ M. The response of the conventional amperometric device for increasing atrazine concentration and the relative calibration curve are displayed in Figure 5 and Figure 6, respectively.

The main analytical data, obtained using the immunosensor device and based on surface plasmon resonance were compared with those obtained using both conventional and screen-printed immunodevices (Table 1). In this table a detailed comparison was made of the main analytical features of the new SPR devices with those of two other immunosensors, so that the advantages and disadvantages of the new SPR method could be evaluated. The linear range of the screen-printed and conventional devices was about three and five decades, respectively, and the LOD values about 10^−8^ M and 5 × 10^−11^ M, respectively. The linear range of the SPR device was just over one decade larger and the LOD of the same order of magnitude as that of the conventional immunosensor, although higher than that of the screen-printed device. Therefore, the most attractive aspect of the method using the SPR device was the measurement time, which was found to be about half that required in the case of the two competitive methods.

Using SPR experiment it was also possible to obtain important parameters about the kinetica of the antigen-antibody interaction. The SPR signal, during the binding process, expressed as a function of time, contains kinetic information and this allowed a full kinetic characterization of the interaction between atrazine and anti-atrazine. The binding reaction equation of antigen (A) and antibody (B) for one-to-one binding reaction can be written as:

A + B ↔ AB with K_D_ = k_d_/k_a_ = [A][B]/[AB]

where k_a_ is the association rate constant in M^−1^·s^−1^ and k_d_ is the dissociation rate constant in s^−1^. K_D_ is the equilibrium dissociation constant and in this study it was found to be 0.48 ± 0.5 M. Based on the equilibrium dissociation constant K_D_ and the dissociation rate, the association rate constant can be calculated as k_a_ = k_d_/K_D_. Using nonlinear fitting of the data in the analytical response at equilibrium as a function of concentration, the k_d_ can be calculated. In this study, the value of k_a_ was found to be 7.5 ± 0.6 M^−1^ s^−1^ and k_d_ was found to be 3.59 ± 0.5 s^−1^, respectively.

**Table 1:** Analytical characterization of immunosensor method for atrazine determination, by SPR, conventional and screen-printed immunodevices using the direct method in the first case and competition procedure in the last two cases.

Methods	Atrazine Determination by Means of SPR Immunosensor	Atrazine Determination by Means of Conventional Immunosensor	Atrazine Determination by Means of Screen-Printed Immunosensor
Regression equation (Y = a.u., X = M)	y = 121.0 (±6.4) + 0.8 (± 0.5)	y = 34.1 (±0.8) log x + 408.8 (±6.2)	y = 10.9 (±1.3) log x + 31.2 (±0.6)
level of confidence (1 − α) = 0.95;	(n − *ν*) = 7; (t = 2.12)	(n − v) = 16; (t = 2.12)	(n − *v*) = 8; (t = 2.78)
Linear range (M)	1.0 × 10^−7^–1.5 × 10^−6^	1.0 × 10^−10^–2.8 × 10^−5^	5.0 × 10^−8^–2.0 × 10^−5^
Correlation coefficient (*R*^2^)	0.9857	0.9891	0.9736
Repeatability of the measurement (as pooled SD %)	5.5	5.3	7.2
Low detection limit (LOD) (M)	5.3 × 10^−8^	5.0 × 10^−11^	2.3 × 10^−8^
Analysis time	≈1 h	≈1 h	≈25 min

In Table 2 a comparison between the selectivity toward several triazine and non triazine pesticides both for the conventional and the screen-printed and SPR immunosensors is shown. The chemical structure of several compounds belonging to the triazine pesticide group is very similar (several triazine pesticides have structural formulae similar to that of the atrazine), so that considering the selectivity of SPR device, the atrazine antibody used in the present research displays the best reactivity to atrazine (100%), but also a considerable level of reactivity toward simazine (≈90%), atrazine-desethyl (≈40%) and a little lower toward azinphos-ethyl (≈15%), whereas toward pesticides of the carbamate group such as carbaryl and aldicarb, it was only about 12%. Lastly, the reactivity toward pesticides with a completely different structure, such as 2,4-D, was almost negligible, as may be inferred from the data also set out in Table 2. 

**Table 2:** Comparison of selectivity, (%) response to different pesticides both by SPR method and conventional or screen-printed immunosensors. Response to atrazine was taken as 100%.

Compound	% Response of SPR Device	% Response of Conventional Immunosensor	% Response of Screen-Printed Immunosensor
Atrazine	100.0	100.0	100.0
Simazine	90.0	87.5	85.0
Atrazine-desethyl	40.0	42.6	/
Azinphos-ethyl	15.0	9.7	12.8
2,4-D	8.0	28.0	20.0
Carbaryl	5.0	3.0	8.5
Aldicarb	5.0	2.0	/

The same table also shows how selectivity towards pesticides of the triazine class does not differ appreciably in the case of the other immunosensors, which are however less selective toward 2,4-D (reactivity in this case is of the order of 20%–28%). On the other hand, the k_aff_ values obtained using the conventional, screen-printed and SPR immunosensor methods (see Table 3) are in all cases of the order of 10^6^ M^−1^, that is, comparable in value.

**Table 3:** IC_50_ and k_aff_ values obtained using three different immunosensors.

Method	IC_50_ *n* = 5; RSD% ≤ 5 (M)	k_aff_ *n* = 5; RSD% ≤ 5 (M^−1^)
Surface plasmon resonance in flow	8.0 × 10^−7^	1.25 × 10^6^
Conventional immunosensor	6.5 × 10^−7^	1.54 × 10^6^
Screen printed immunosensor	1.0 × 10^−6^	1.00 × 10^6^

**Figure 7:**
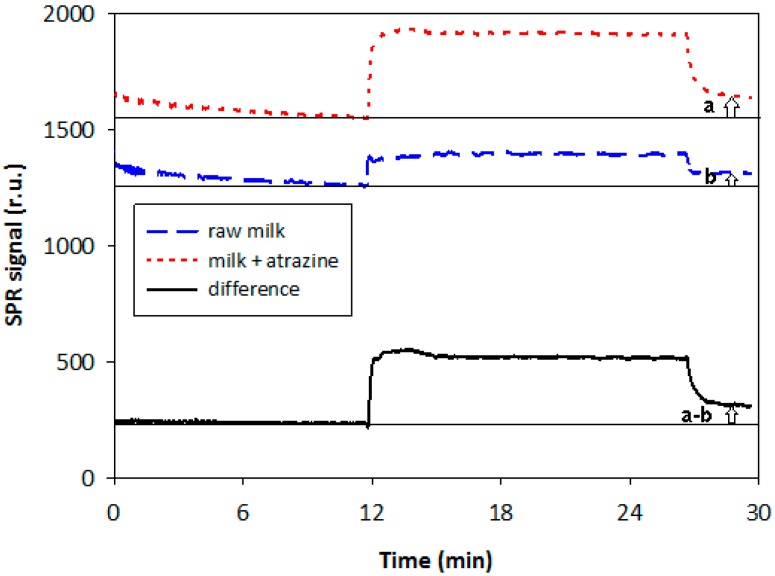
Typical measurement recorded for the detection of atrazine in bovine milk by SPR device. Arrows indicated the measurement format.

Lastly some applications and comparisons were carried out on real samples, checking for triazine pesticides in bovine milk samples. In Figure 7, a typical measurement recorded for the detection of atrazine in bovine milk by SPR detection is reported as an example. The dashed curve and the dotted curve were obtained simultaneously using the two-channel BioSuplar 400, running suitably diluted raw milk through the first channel (dotted curve) and milk fortified with atrazine through the second channel (dashed curve); the unbroken curve represents the difference between the two signals. As can be seen, after stabilization for about 10 min, the sample addition produces a signal increase in both cases but which is much more pronounced for the fortified sample. The successive washing with buffer leads to a signal decrease down to baseline only in the case of raw milk, which indicates the absence of atrazine in the raw sample, while in the case of the fortified sample the signal attains a constant value higher than the baseline, as expected, taking into account that atrazine stably binds to the antibody immobilized on the sensor chip; this enables this pesticide to be detected and quantified in the real sample.

Six different commercial bovine samples were analyzed for triazinic pesticides concentration using the SPR immunosensor method. In all cases the triazine pesticide concentration level was found lower than the LOD of the method, *i.e.*, 5 × 10^−8^ M, or 5 × 10^−11^ M, according by the used methods.

The absence of triazine pesticides at a concentration higher than 5 × 10^−8^ M in all the commercial milk samples tested was also corroborated by the measurements performed using the other two immunosensors.

Several recovery tests, performed on bovine milk samples using both SPR and conventional or screen-printed amperometric immunosensors performed on all six milk samples, are reported in Table 4, Table 5 and Table 6. Recovery tests were first carried out on the same bovine milk samples spiked only with atrazine. The results of experimental atrazine “recovery” tests obtained using the SPR device are summarized in Table 4, while the results of several different triazinic pesticide “recovery” tests were reported in Table 5.

**Table 4:** Atrazine recoveries from spiked samples of bovine milk using the SPR immunosensor and the direct method.

Milk Sample Number	Found Atrazine Concentration (M) (*n* ≥ 3)	Pesticide Added in Bovine Milk	Total Concentration Value after Spiking (M)	Experimental Conc. Value (M) ± SD (*n* ≥ 3)	% Recovery
1	<5.0 × 10^−8^	Atrazine	1.89 × 10^−7^	(1.89 ± 0.03) × 10^−7^	100.0%
2	<5.0 × 10^−8^	Atrazine	1.89 × 10^−7^	(2.01 ± 0.02) × 10^−7^	103.3%
3	<5.0 × 10^−8^	Atrazine	2.64 × 10^−7^	(2.40 ± 0.03) × 10^−7^	94.9%

**Table 5:** Recovery data of different triazine pesticides from spiked samples of bovine milk using the SPR immunosensor and the direct method.

Milk Sample Number	Found Pesticide Concentration (M) (*n* ≥ 3)	Pesticide Added in Bovine Milk	Total Concentration Value after Spiking (M)	Experimental Conc. Value (M) ± SD (*n* ≥ 3)	% Recovery
4	<5.0 × 10^−8^	Simazine	3.00 × 10^−7^	(2.85 ± 0.03) × 10^−7^	95.8
5	<5.0 × 10^−8^	Atrazine-desethyl	3.00 × 10^−7^	(2.79 ± 0.02) × 10^−7^	93.0
6	<5.0 × 10^−8^	Azinphos-ethyl	3.00 × 10^−7^	(2.73 ± 0.03) × 10^−7^	92.6

**Table 6:** Some pesticide recoveries from spiked samples of bovine milk using the conventional (a) and screen-printed (b) amperometric immunosensors and the competitive format illustrated in Figure 3.

Milk Sample Number	Method	Found Pesticide Concentration (M) (n ≥ 3)	Pesticide Added in Bovine Milk	Total Conc. Value after Spiking (M)	Experimental Conc. Value (M) ± SD (n ≥ 3)	% Recovery
1	(a)	<5.0 × 10^−11^	Atrazine	10.0 × 10^−8^	(9.8 ± 0.10) × 10^−8^	98.5%
2	(a)	<5.0 × 10^−11^	Simazine	10.0 × 10^−8^	(9.7 ± 0.12) × 10^−8^	97.3%
3	(a)	<5.0 ×10^−11^	Atrazine-desethyl	10.0 × 10^−8^	(9.4 ± 0.25) × 10^−8^	94.4%
4	(a)	<5.0 × 10^−11^	Azinphos-ethyl	10.0 × 10^−8^	(9.3 ± 0.10) × 10^−8^	93.3%
5	(b)	<2.5 × 10^−8^	Atrazine	10.0 × 10^−8^	(9.6 ± 0.10) × 10^−8^	96.5%
6	(b)	<2.5 × 10^−8^	Simazine	10.0 × 10^−8^	(9.8 ± 0.13) × 10^−8^	98.3%

Examination of the data obtained indicates that the average recovery generally lies between 103.3% and 94.9% for the atrazine, and 95.8% and 92.6% for other triazinic pesticides in the samples tested. Recovery tests involving the same triazine pesticides were then carried out on the same bovine milk samples but using the conventional and screen-printed immunosensors. Results are summarized in Table 6. In short, satisfactory results were obtained in all the tests; indeed the data obtained indicate that the average recovery is generally between 98.5% and 93.3% for all the triazine pesticides in the samples tested.

## 4. Materials and Methods

### 4.1. Samples

All six different samples of commercial fresh bovine milk were purchased in a local supermarket and analyzed without treatment, except (where necessary) for possible dilution with distilled deionized water.

### 4.2. Reagents and Materials

Anti-atrazine monoclonal antibody and atrazine carboxy derivatives were provided by Eremin (Department of Chemical Enzymology, Faculty of Chemistry, Moscow State University, Russia). 1-Chloro-3-ethylamino-5-isopropylamino-2,4,6-triazine (atrazine), 2-chloro-4-ethylamino-6-isopropyl-amino-1,3,5-triazine (atrazine-desethyl), 6-chloro-*N*,*N*’-diethyl-1,3,5-triazine-2,4-diamine (simazine), 3-(diethoxyphosphinothioylsulfanylmethyl)-1,2,3-benzotriazin-4-one (*i.e.*, azinphos-ethyl), 2-methyl-2-(methylthio)propanal (aldicarb) and 1-naphthylmethylcarbamate (or carbaryl) were provided by Pestanal Sigma-Aldrich (St. Louis, MO, USA). Potassium chloride, hydrogen peroxide 30% (w/v); dibasic and monobasic anhydrous potassium phosphate RPE, chloroform PE, dichloromethane RPE and diethylether RPE were supplied by Carlo Erba Reagents (Milan, Italy). Ny^+^ Immobilon Affinity membrane (porosity 0.65 µm) was provided by the Millipore Corporation (catalog number INYC08550; New York, NY, USA).

### 4.3. Materials for SPR Measurements

Monobasic sodium phosphate, dibasic sodium phosphate, potassium chloride, sodium chloride, 1,1-mercaptoundecanoic acid (MUA) (95%), *N*-(3-dimethylaminopropyl)-*N*'-ethylcarbodiimide hydrochloride (EDC, commercial grade), *N*-hydroxysuccinimide (NHS, 98%) were purchased from Sigma-Aldrich. All solutions were prepared using ultrapure deionized water (resistance: 18.2 mΩ × cm at 25 °C; TOC < 10 g·mL^−1^) obtained using a Direct-Q UV3 Merck Millipore system (Billerica, MA, USA).

### 4.4. Apparatus for SPR Measurements

The Sensordisc AU bare gold disks for SPR analysis was purchased from XanTec Bioanalytics GmbH (Duesseldorf, Germany). For the SPR measurements, performed using the flow operating mode (see Figure 1), a BioSuplar 400T (Analytical µ-Systems—Dep. of Mivitec GmbH, Sinzing, Germany), was used.

### 4.5. Apparatus for Conventional and Screen-Printed Immunosensors

For triazine pesticide analysis a mod. 551 VA­Detector Amel srl (Milan, Italy) potentiostat was used connected to an amperometric hydrogen peroxide electrode from Universal Sensor Inc., (New Orleans, LA, USA), Mod. 4006a and to a mod. d5126­2 Omniscribe (by Bausch & Lomb Incorporated, Rochester, UK) analog recorder. The test solution was contained in a thermostated cell at 23 °C and under constant magnetic stirring by an Amel srl mod. 291/lf apparatus.

For the amperometric screen printed measurements a wafer made of corundum ceramic was used as support and both working and counter electrodes were made of platinum. A Pt working electrode was modified with electrodeposited Prussian Blue, thus enabling H_2_O_2_ amperometric detection when polarized at 0 mV *vs.* Ag/AgCl/Cl^−^ reference electrode. At the end of the sensor there was a contacting field connected to the active part by silver conducting paths, which were covered by a dielectric protection layer, while the bio-chemically active antibody immobilized on the Immobilon membrane was fixed to the working electrode of the sensor. The response of the screen-printed device increasing atrazine concentration was recorded.

For screen­printed measurements an amperometric screen­printed transducer, fabricated as shown in Figure 2b and with Pt working electrode modified by electrodeposited Prussian Blue polarized at +0.0 mV *vs.* reference electrode Ag/AgCl/Cl^−^, was purchased from BVT Technologies (Praha, Czech Republic), and connected for the measurements to a PalmSens Electrochemical Interface (PalmSens BV, Utrecht, The Netherlands). The hydrogen peroxide screen-printed electrode employed was modified using an Immobilon membrane (containing the immobilized anti-atrazine antibody) in close contact with the surface of the electrode itself [25,26].

### 4.6. Biotinylation Materials

The biotinylation kit, supplied by Sigma Immunochemicals (St. Louis, MO, USA), was composed of biotinylation Reagent (BAC-SulfoNHS, namely biotinamido hexanoic acid 3-sulfo-N-hydroxysuccinide ester), 5 M sodium chloride solution, micro-spin column (2 mL; in practice, a small empty cylindrical vessel prepackaged with Sephadex G-50), 0.1 M sodium phosphate buffer pH 7.2, 0.01 M phosphate buffer saline (PBS) pH 7.4 (reconstituted with 1 L of deionized water to give 0.01 M phosphate buffer, 0.138 M NaCl, 2.7 mM KCl, pH 7.4); and ExtrAvidin^®^ peroxidase (containing 0.2 mL of ExtrAvidin Peroxidase conjugate at 2.0 mg·mL^−1^, supplied with 0.01% thimerosal). Dialysis membrane (art. D-9777), 1-ethyl-3 (3-dimethylaminopropyl) carbodiimide, albumin (from bovine serum) (BSA) and TRIS (hydroxymethyl-aminomethane) and TWEEN^®^ 20 provided by Sigma Aldrich.

## 5. Conclusions

The calibration curve for atrazine using the proposed SPR device displayed a linearity range from 1.0 × 10^−7^ to 1.5 × 10^−6^ M and an LOD value of about 5.0 × 10^−8^ M. The linear range of the screen-printed and conventional devices was about three and five decades, respectively, with LOD values of about 10^−8^ M and 5.0 × 10^−11^ M, respectively, although the measurement time using the SPR device was found to be about half that required in the case of the two competitive methods. Table 2 shows that the k_aff_ values were found to be about the same (*i.e.*, of the order of 10^6^ M^−1^); also the selectivity toward several triazine pesticides was about the same using both SPR and conventional or screen-printed immunosensor methods, although it must be highlighted that the SPR device had a better selectivity toward other non-triazine pesticides. Recovery tests performed on bovine milk samples and reported in Table 4, Table 5 and Table 6 confirmed that new surface plasmon resonance, as well as both the conventional or screen-printed immunosensor method, is suitable for the analysis of triazine pesticides in milk samples with the advantage that the SPR measurements require about the half the time of the conventional immunosensor method.
